# Simultaneous adrenal and extra-adrenal myelolipoma – an uncommon incident: case report and review of the literature

**DOI:** 10.1186/1477-7819-6-72

**Published:** 2008-07-04

**Authors:** Derek Zieker, Ingmar Königsrainer, Stephan Miller, Ulrich Vogel, Karl Sotlar, Wolfgang Steurer, Alfred Königsrainer, Thorsten G Lehmann

**Affiliations:** 1 Department of General, Visceral and Transplant Surgery, Comprehensive Cancer Center, Tuebingen, Germany; 2 Department of Transfusion Medicine, Comprehensive Cancer Center, Tuebingen, Germany; 3 Department of Radiology, Comprehensive Cancer Center, Tuebingen, Germany; 4 Department of Pathology, Comprehensive Cancer Center, Tuebingen, Germany

## Abstract

**Background:**

Extra-adrenal myelolipomas are rare benign tumours. Other soft tissue tumours such as well-differentiated liposarcomas appear morphological almost identical. Preoperative imaging and especially biopsy are important tools to diagnose these lesions.

**Case presentation:**

We report a very seldom case of a simultaneous myelolipoma of the adrenal gland in association with an extra-adrenal myelolipoma in an 75-year-old man. With a review of the literature we describe and discuss the aetiology, differential diagnosis and treatment of patients with respect to adrenal and extra-adrenal lesions.

**Conclusion:**

The appearance of a simultaneous adrenal and extra-adrenal myelolipoma is a rare incident. We conclude that such lesions should be considered in the differential diagnosis of a fat-containing tumour in the retroperitoneal tissue/compartment.

## Background

The incidence of extra-adrenal myelolipomas is rare. Only about 50 cases have been described in the literature within the last 2 decades. Myelolipomas are benign tumours and are composed of haematopoietic cells and adipose tissue [1-4]. They are usually non-functioning asymptomatic tumours and often found incidentally on radiographic studies [5]. Mostly myelolipomas are located in the adrenal gland. A very infrequent finding is the incidence of a myelolipoma of the adrenal gland simultaneously with an extra-adrenal myelolipoma. Appearance of myelolipomas outside of the adrenal gland might be difficult to identify, since other soft tissue tumours such as well-differentiated liposarcomas appear morphological almost identical [1-3,6-10]. We report an unusual case of a myelolipoma of the adrenal gland in association with an extra-adrenal myelolipoma. This case sensitises the importance of this combination as a pitfall in the correct diagnosis and management of patients with respect to adrenal and extra-adrenal lesions.

## Case presentation

**Figure 1:**
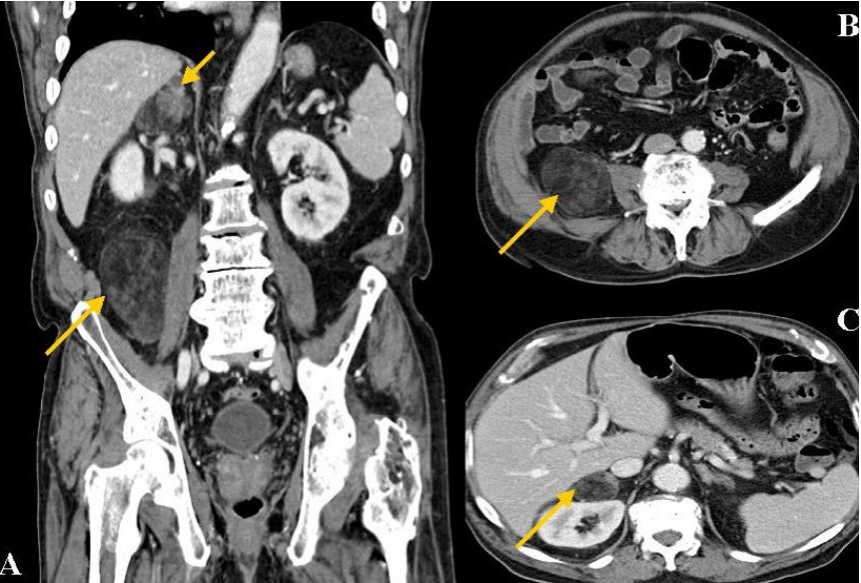
**CT imaging of the abdomen**. **A: **Coronal abdominal CT scan detected two fat-containing masses in the adrenal gland and lateral of the psoas muscle inferior to the right kidney. Both lesions appeared identical in the CT imaging and were at first consistent with a well-differentiated liposarcoma. Intra-operative biopsy followed by rapid section revealed a simultaneous adrenal and extra-adrenal myelolipoma. **B and C: **Further CT images of the extra-adrenal/retorperitoneal myelolipoma.

A 75-year-old man with a history of persisting abdominal pain and mild diarrhoea for three months was referred by a general practitioner to the hospital. During this period the patient observed a weight loss of 2 kg, but did not show any B symptoms. A colonoscopy was performed without pathological findings. A subsequent CT examination of the abdomen showed two separate fat-containing retroperitoneal masses one in the adrenal gland and the second lateral of the psoas muscle inferior to the right kidney, outside the peri-renal adipose tissue. The lesions were separate and had no connecting tissue in between them (Figure 1). To further confirm the obtained CT scan results an MR imaging was performed and showed again a fat-containing lesion in the adrenal gland and a 7 × 5 × 7 cm soft tissue-tumour inferior to the right kidney without invasion of the right kidney, urethra or renal vasculature. Both lesions appeared similar in the CT and MR imaging and were consistent with a well-differentiated liposarcoma (Figure 1). Aware of the fact that a liposarcoma of the adrenal gland is extremely rare, a primary benign and fat containing tumour derived from the adrenal gland itself, such as a lipoma or myelolipoma was considered. Regarding tumour markers, only increased levels of CA 19-9 were detected (81.86 U/ml reference < 37 U/ml, CEA 2.2 μg/l, reference < 5 μg/l). To exclude a neuroendocrinological pathology additional tests were performed but did not reveal any noticeable conspicuities.

Consequently, we assumed a combination of a retroperitoneal liposarcoma and a primary benign, lipomatous tumour of the adrenal gland. Alternatively, the very rare case of the combination of a retroperitoneal liposarcoma and an adrenal liposarcoma was taken into consideration. Therefore, two therapeutic options were considered on these accounts. In the unlikely case of simultaneous liposarcomas an *enbloc *resection including both masses would be required after pre-treatment. But, in the probable situation of a benign lipomatous primary lesion of the adrenal gland in combination with a retroperitoneal liposarcoma an adrenalectomy with an additive biopsy of the infrarenal mass was decided as the strategy of choice in this individual case.

Concerning this strategy a neoadjuvant radiotherapy followed by radical surgery, preventing the kidney, could have been performed as it has been approved as follows. To perform a neoadjuvant therapy a positive biopsy for sarcoma would be a compulsory prerequisite. Prognostic survival markers for retroperitoneal liposarcomas are the histologic subtype and margin of resection [11]. Response rates to neoadjuvant chemotherapy alone are less than 10% [11]. Preoperative radiation therapy or combined radio-chemotherapy with consecutive radical resection improves survival [12,13]. Concerning surgery complete resection of the lesion is to be achieved. Extended resection including contiguous organs without pre-treatment is associated with an increased risk without influence on disease specific survival and is therefore considered to be inappropriate [11].

According to our described strategy, the patient received a laparotomy. As expected, the adrenal tumour appeared well-encapsulated in accordance with a benign lipomatous tumour. Following the above mentioned second option first an adrenalectomy was performed. The rapid section of the adrenal lesion revealed the result of a myelolipoma. Having in mind that both lesions presented morphologically identical in CT and MR imaging and the assessed intraoperatively analogousness of the masses, we resigned a biopsy of the infrarenal tumour and removed it *in toto *without the kidney. Once more the rapid section revealed a myelolipoma. The final histology described a 4 cm sized and in weight 46 g well-encapsulated myelolipoma of the adrenal gland. Further a 14 cm sized and in weight 250 g well-encapsulated retroperitoneal myelolipoma was determined, without capsule involvement. Both tumours were composed of predominantly mature adipose tissue with mature myeloid elements, allowing the diagnosis of an extra-adrenal and adrenal myelolipoma. Consequently no other treatment than surgery was indicated and has not been performed in this case.

Postoperatively, the patient's recovery was uneventful and he was subsequently discharged.

## Discussion

Mostly myelolipomas present in the adrenal gland and are well-circumscribed lesions that contain mature adipose tissue intermixed with mature myeloid elements. Only about 50 cases of extra-adrenal myelolipomas were reported yet in the literature [3]. The occurrence of most extra-adrenal myelolipomas were noted in the presacral soft tissue, followed by the retroperitoneum, the pelvis the stomach and in the musclefascial as well as a few have been reported in the perirenal tissue [1,3,4,8,9,14-18]. The aetiology of myelolipomas in general is so far unknown, although derivation from bone marrow tissue is discussed [2]. A further potential hypothesis is that adrenal myelolipomas emerge from metaplasia either of previously uncommitted adrenal cortical mesenchymal cells or of during intrauterine life migrated haematopoietic stem cells [1,3]. Preferential myelolipomas occur in females more often than in males and more often in middle-aged to the elderly [15-17]. Typically myelolipomas of the adrenal and extra-adrenal gland are asymptomatic but larger lesions can cause symptoms from mass effect or haemorrhage [15-17,19]. Malignant degeneration of myelolipomas has not been reported so far [2]. Since myelolipomas are often asymptomatic their detection via CT or MR imaging are mostly incidentally findings [2,4-6,9,10,14,18].

The radiographic diagnosis of an adrenal and extra-adrenal myelolipoma preoperatively is rather a challenge. Appearing as a fat-containing tumour a differentiation between an adrenal or extra-adrenal myelolipoma and other fat-containing retroperitoneal tumours can be difficult. The majority of fat-containing tumours are well-differentiated liposarcomas [20,21]. Since extra adrenal myelolipomas are histologically identical to their adrenal counterpart, the CT and MR findings should appear similar [2,22]. A fact that was proven in our case since both lesions appeared almost identical in the CT imaging (see Figure 1). Concerning the described case, at first an infrarenal metastasis or an adrenal and extra-adrenal liposarcoma was assumed.

**Figure 2:**
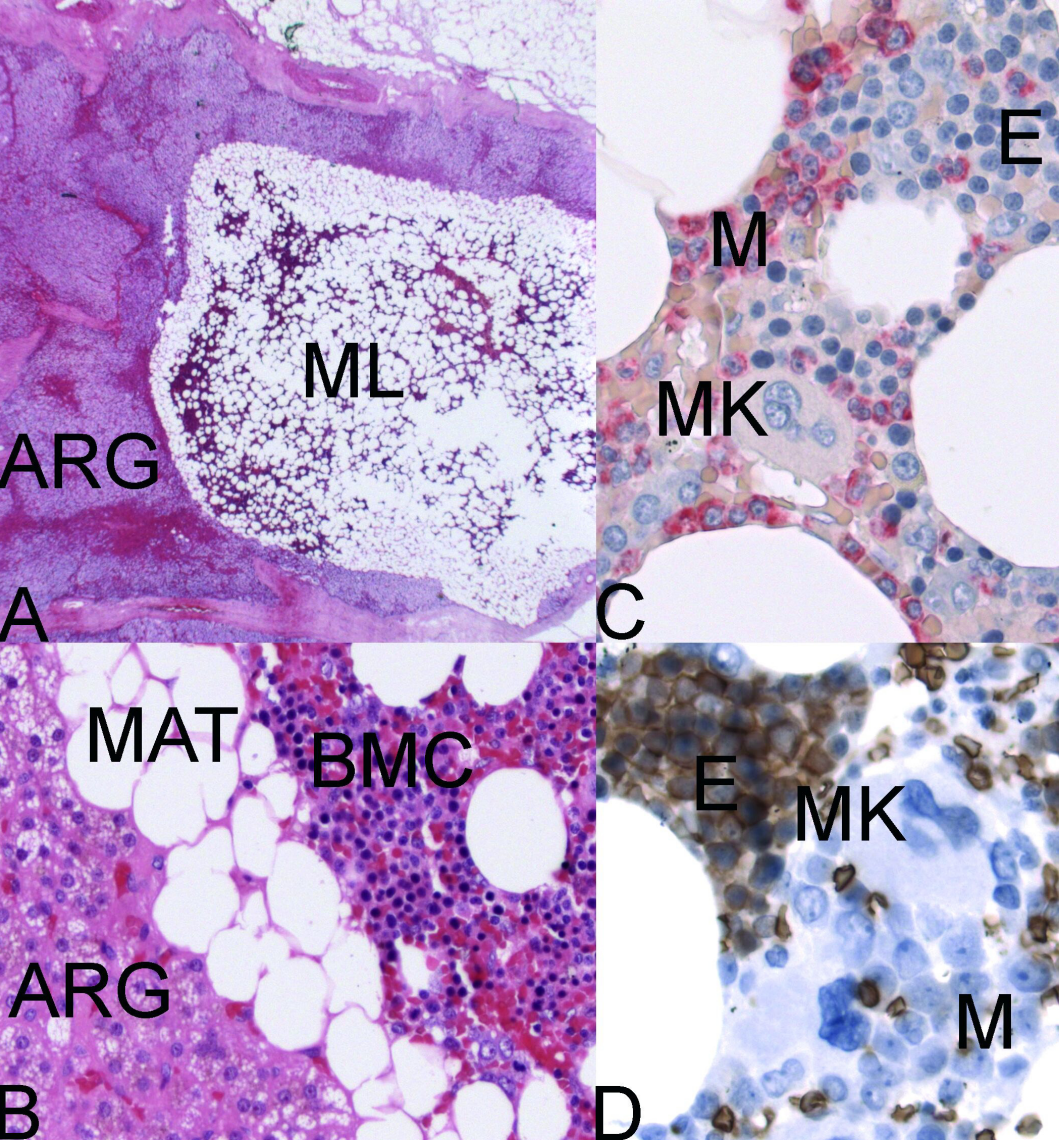
**Myelolipoma**. **A: **Adrenal gland with intraparenchymal myelolipoma (Hematoxylin-Eosin, ×12.5) ARG = adrenal gland; ML = myelolipoma. **B: **Adrenal cortical cells with foamy cytoplasm, mature adipose tissue and bone marrow cells (Hematoxylin-Eosin, ×200). **C: **Mature adipose tissue and bone marrow cells (Naphthol-AS-D chloroacetate esterase (Leder), ×400); E = erythroid; M = myeloid (red); MK = megakaryocyte. **D: **Mature adipose tissue and bone marrow cells (Immunohistochemistry, anti-Glycophorin-A, Diaminobenzidine (DAB), ×400); E = erythroid (brown); M = myeloid; MK = megakaryocyte.

Histologically, extra adrenal myelolipomas can be readily differentiated from other entities [20,21]. Myelolipomas have to be distinguished from mass-forming foci of extramedullary haematopoiesis such as myeloproliferative diseases, haemolytic anaemia and severe skeletal disease. These extramedullary haematopoietic "tumours" lack fat and are ill defined. Unlike extra-adrenal myelolipomas that are usually well encapsulated and composed of variable amounts of mature adipose tissue, smooth muscle and bone marrow cells, liposarcomas tend to be poorly marginated, not hemorrhagic have lipoblasts and zones of cellular atypia [2,21]. A pathologic challenge is the differentiation of extra adrenal myelolipomas from other processes that contain haematopoietic tissue and mature adipocytes. Mesenchymal tumours and teratomas may contain some of these elements but also contain other tissue subtypes as well [2]. In our case, bone marrow, mature adipose tissue was evident microscopically but other tissue subtypes did not appear (Figure 2).

A major pitfall is an overlooked diagnosis of a low-grade liposarcoma resulting in diametrically opposite consequences, concerning treatment and prognosis. For example in case of a liposarcoma neoadjuvant treatment seems to be the therapy of choice and not primary extensive surgery.

In our case the intraoperative detection of the macroscopically benign and encapsulated lesions, the pre-operative identical CT and MR imaging of both tumours followed by intra-operative rapid section of the adrenal tissue, prevented us from a radical *en bloc *resection or even from a second operation to remove the retroperitoneal mass after pre-treatment.

## Conclusion

Nevertheless, the appearance of a simultaneous adrenal and extra-adrenal myelolipoma is a rare incident. Therefore, we conclude that such lesions should be considered in the differential diagnosis of a fat-containing tumour in the retroperitoneal tissue/compartment. Summarized, this case underlines the importance of pre-operative CT and MR imaging, intra-operative rapid section and the importance of the surgeons' intraoperative judgement of the tumour, concerning an unknown primary tumour located in the adrenal gland and the retroperitoneum.

## Competing interests

The authors declare that they have no competing interests.

## Authors' contributions

DZ and IK drafted the article. SM carried out the radiologic work up and helped in drafting the article. UV carried out the histological work up and helped in drafting the article. KS carried out the histological work up and helped in drafting the article. WS carried out surgery and helped in drafting the article. AK carried out surgery, conceived of the study and participated in its design and coordination and helped to draft the article. TL carried out surgery, supervised the preparation of the article and helped preparing the final manuscript.
